# Supplementary material for: Vasoactive intestinal peptide controls the suprachiasmatic circadian clock network via ERK1/2 and DUSP4 signalling
Source: Nat Commun. 2019 Feb 1;10:542. doi: 10.1038/s41467-019-08427-3 (PMC6358603; doi:10.1038/s41467-019-08427-3)
Supplement: Supplementary file 1 — Supplementary Information [file 41467_2019_8427_MOESM1_ESM.pdf]

Vasoactive intestinal peptide controls the suprachiasmatic circadian clock network via ERK1/2 and DUSP4 signalling

Hamnett et al.

Supplementary Figure 1

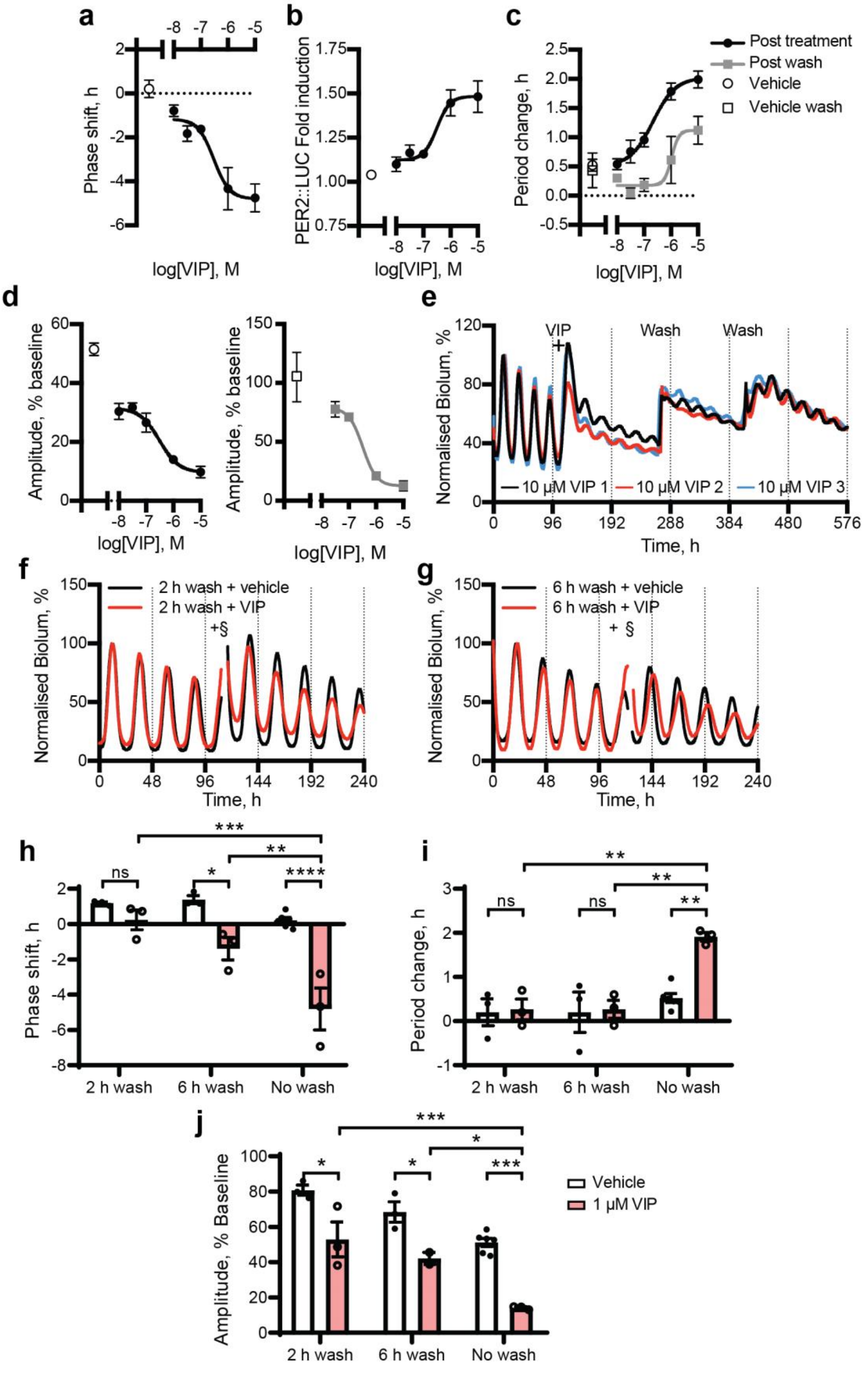

Supplementary Figure 1 | VIP effects on SCN slice rhythmicity are dose- and duration-dependent (**a-d**) Dose-dependent responses in phase shifts (**a**), PER2::LUC fold induction (**b**), period change (**c**) and relative amplitude change (**d**) (mean ± SEM) following treatment with VIP. Grey squares in (**c,d**) represent values following media change. Data were fitted with a nonlinear regression. (**e**) Representative PER2::LUC bioluminescence rhythms of 3 SCN slices treated with 10 µM VIP, followed by two media changes approximately 7 and 13 days after VIP treatment. (**f,g**) Representative PER2::LUC bioluminescence rhythms of SCN slices treated with 1 µM VIP or vehicle at CT10 and washed off after 2 h (**f**) or after 6 h (**g**). Treatments (marked by +) were followed by washing by 4 full media changes (marked by §). Bioluminescence has been normalised to the first peak. (**h-j**) Phase shift (**h**), period change (**i**) and relative amplitude (**j**) responses (mean ± SEM) to VIP followed by wash-off after 2 h (n = 3), 6 h (n = 3) or with no wash (n = 3) with vehicle controls (2 h: n = 3; 6 h: n = 3, no wash: n = 6). All tests two-way ANOVA with Tukey's multiple comparisons test, ns = not significant, $*P < 0.05$, $**P < 0.01$, $***P < 0.001$, $****P < 0.0001$.

Supplementary Figure 2

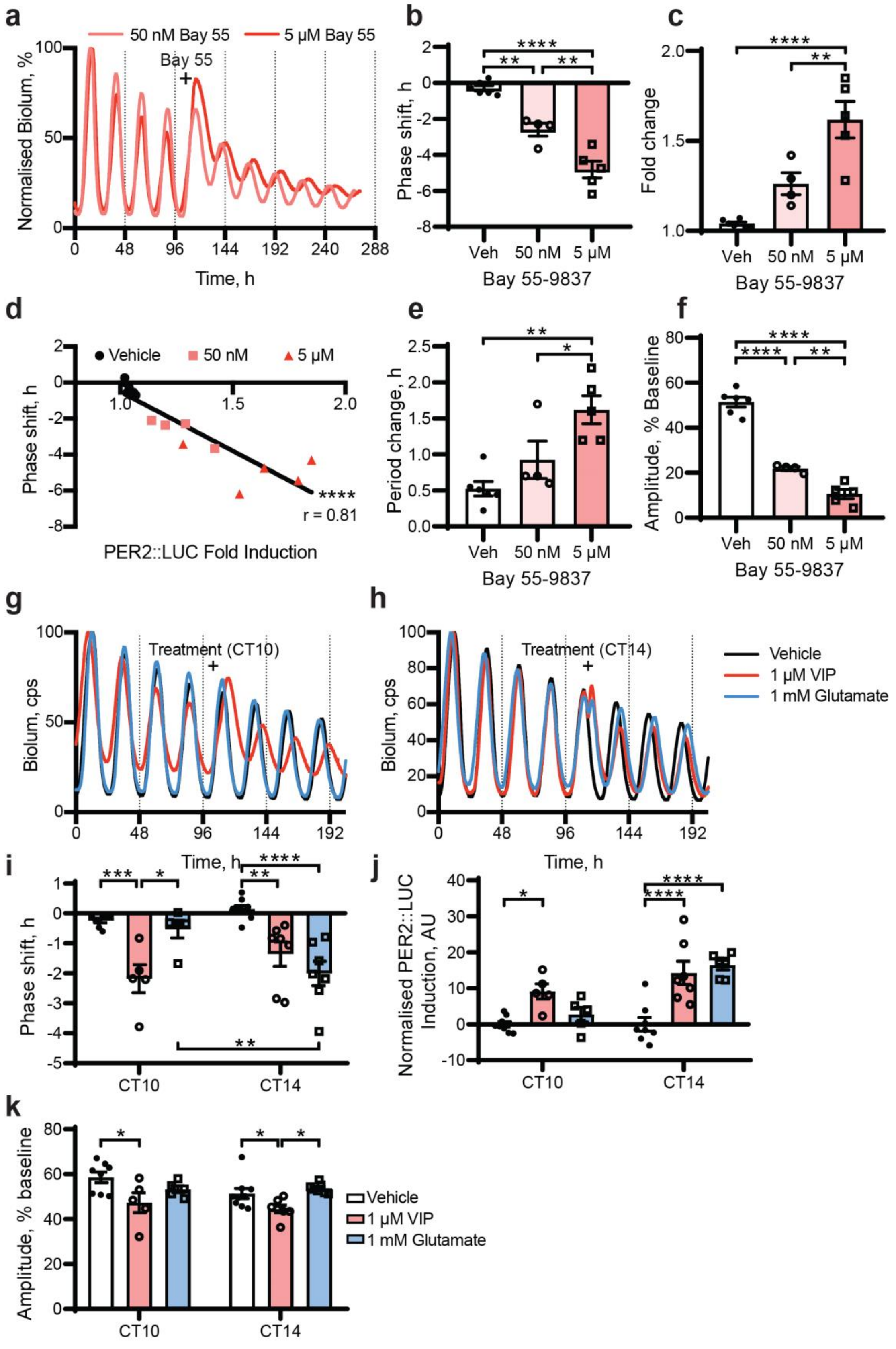

Supplementary Figure 2 | VIP acts through the VPAC2 receptor and produces a distinct response to glutamate. (**a**) Representative PER2::LUC bioluminescence rhythms of SCN slices treated with 50 nM or 5 µM VPAC2 agonist Bay 55-9837 at CT10 (marked by +). Bioluminescence was normalised to the first peak. (**b,c**) Group data for phase shift (**b**) and PER2::LUC fold acute induction (**c**) (mean ± SEM, n = 4, 5, 6 for 50 nM and 5 µM Bay 55-9837, or vehicle respectively. (**d**) Scatterplot of immediate phase shifts vs. PER2::LUC acute induction following Bay 55-9837 treatment. Line represents computed linear regression, r = Pearson's correlation, $^{****}P < 0.0001$. (**e,f**) Group data for period change (**e**), and relative amplitude (**f**) responses (mean ± SEM) to 50 nM or 5 µM Bay 55-9837 compared with vehicle. n as in (**b,c**). (**b,c,e,f**) were analysed with one-way ANOVA with Tukey's multiple comparisons. (**g,h**) Representative PER2::LUC bioluminescence rhythms of SCN slices treated with 1 µM VIP or 1 mM glutamate by direct droplet application at CT10 (**g**) or CT14 (**h**). Bioluminescence was normalised to the first peak. (**i-k**) Phase shift (**i**), PER2 induction (**j**) and relative amplitude (**k**) responses (mean ± SEM) to VIP, glutamate or vehicle at CT10 (n = 5, 5, 8 respectively) or CT14 (n = 7, 7, 9 respectively). PER2 induction (**j**) was normalised by setting mean of vehicle controls to zero. (**i-k**) were analysed with two-way ANOVA with Sidak's multiple comparisons. $^{*}P < 0.05$, $^{**}P < 0.01$, $^{***}P < 0.001$, $^{****}P < 0.0001$.

Supplementary Figure 3

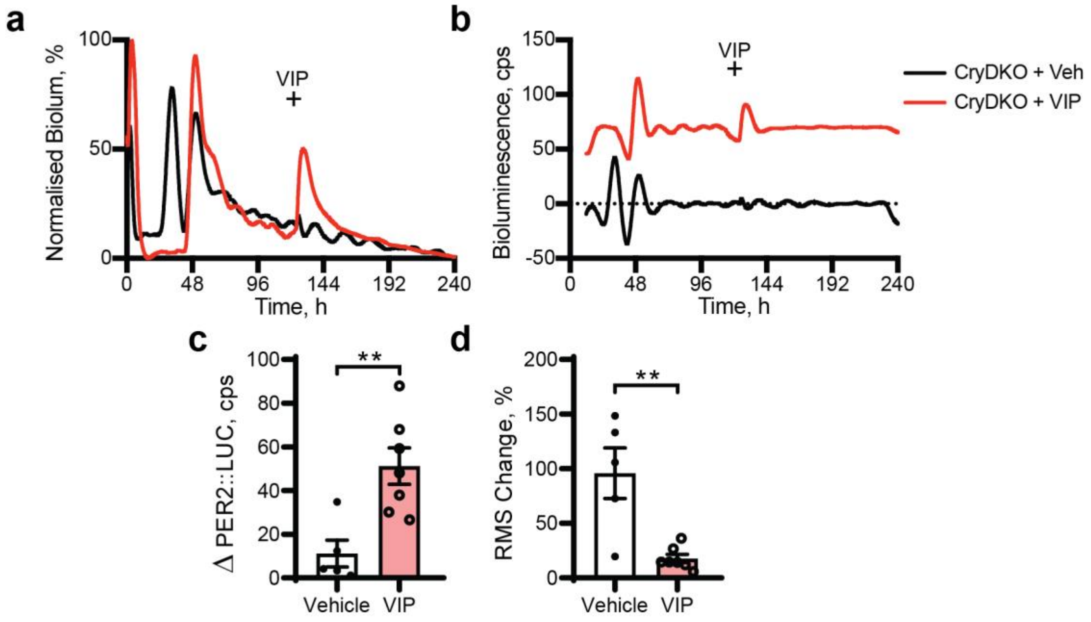

Supplementary Figure 3 | VIP activates PER expression in the SCN independently of the cell-autonomous oscillator. (**a**) Representative PER2::LUC bioluminescence rhythms of *Cry1*$^{-/-}$*Cry2*$^{-/-}$ (CryDKO) SCN slices treated with vehicle or 1 µM VIP (marked by +) at CT10 (phase determined by *Cry1*$^{-/-}$*Cry2*$^{+/-}$ littermate slices, data not shown). Bioluminescence was normalised to the highest value in the recording. (**b**) Detrended (24 h baseline subtraction) PER2::LUC bioluminescence rhythms of slices in (**a**). The VIP-treated slice is offset on the y-axis to aid visualisation. (**c**) Group data for change in levels of PER2::LUC bioluminescence following treatment (n = 5 for vehicle, n = 7 for VIP, mean ± SEM), calculated by subtracting the pre-VIP value from the highest value within the subsequent 6 h recording window. (**d**) Group data for relative root mean square (RMS) change (mean ± SEM) following treatment. RMS was calculated based on data 48 h before and after treatment, omitting a 24 h window immediately after treatment, by taking the square root of averaged normalised, detrended and squared data. All tests unpaired t-tests, $**P < 0.01$.

Supplementary Figure 4

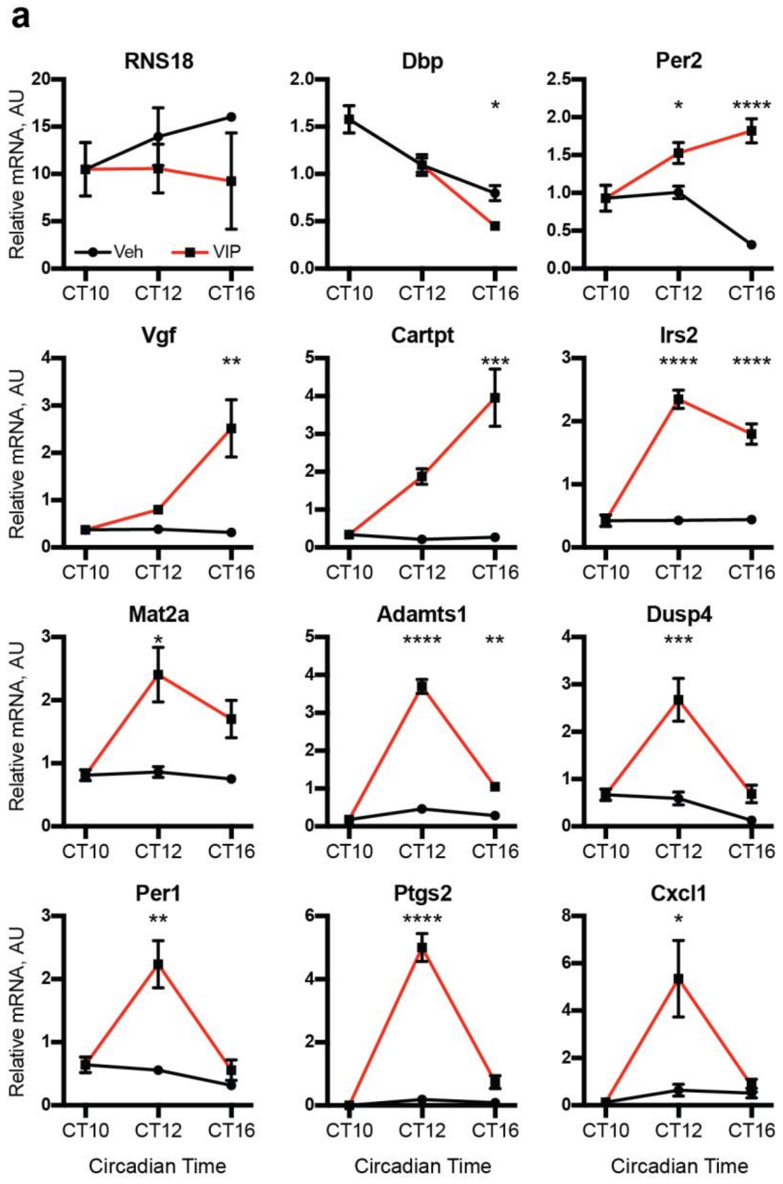

Supplementary Figure 4 | qPCR validation of VIP microarray. (**a**) Relative mRNA expression levels over time and VIP treatment assessed by qPCR (mean ± SEM, n = 3 for CT10, CT12 Veh, CT16 Veh, n = 4 for CT12 VIP and CT16 VIP). All transcript levels except *Rns18* are normalised to *Rns18* quantity to control for total RNA variation. Two way ANOVA with Tukey's multiple comparisons test, *$P < 0.05$, **$P < 0.01$, ***$P < 0.001$ ****$P < 0.0001$.

Supplementary Figure 5

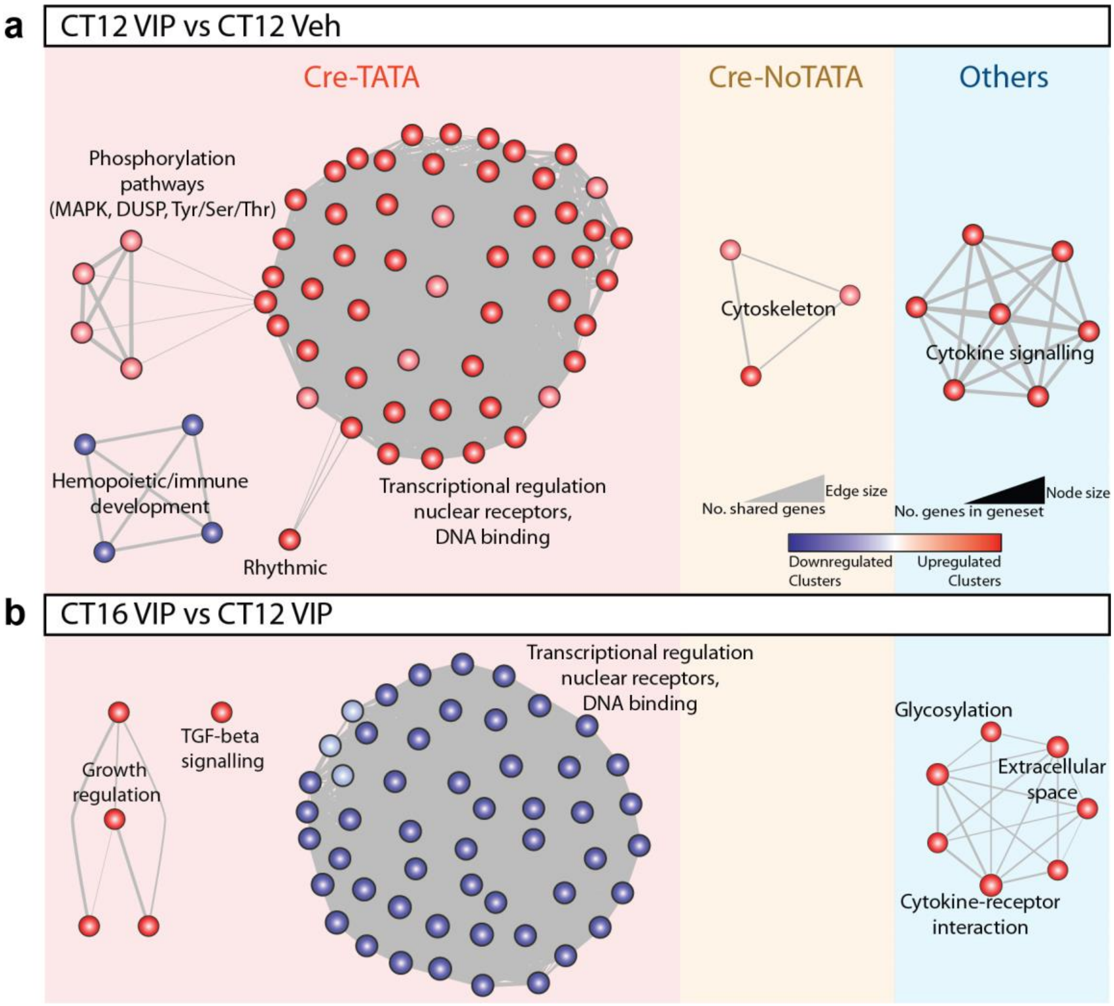

Supplementary Figure 5 | Occurrence of CREs in promoters of VIP-regulated transcripts. (**a,b**) Functional annotation of genes significantly altered by VIP treatment after 2 h (**a**) and after 6 h (**b**) segregated based on promoter elements. CRE-TATA: CRE within 300 bp of TATA box; CRE-NoTATA: CRE in promoter further upstream; Others: No CRE present in promoter (promoter defined as 3 kb upstream to 300 bp downstream of the transcription start site).

Supplementary Figure 6

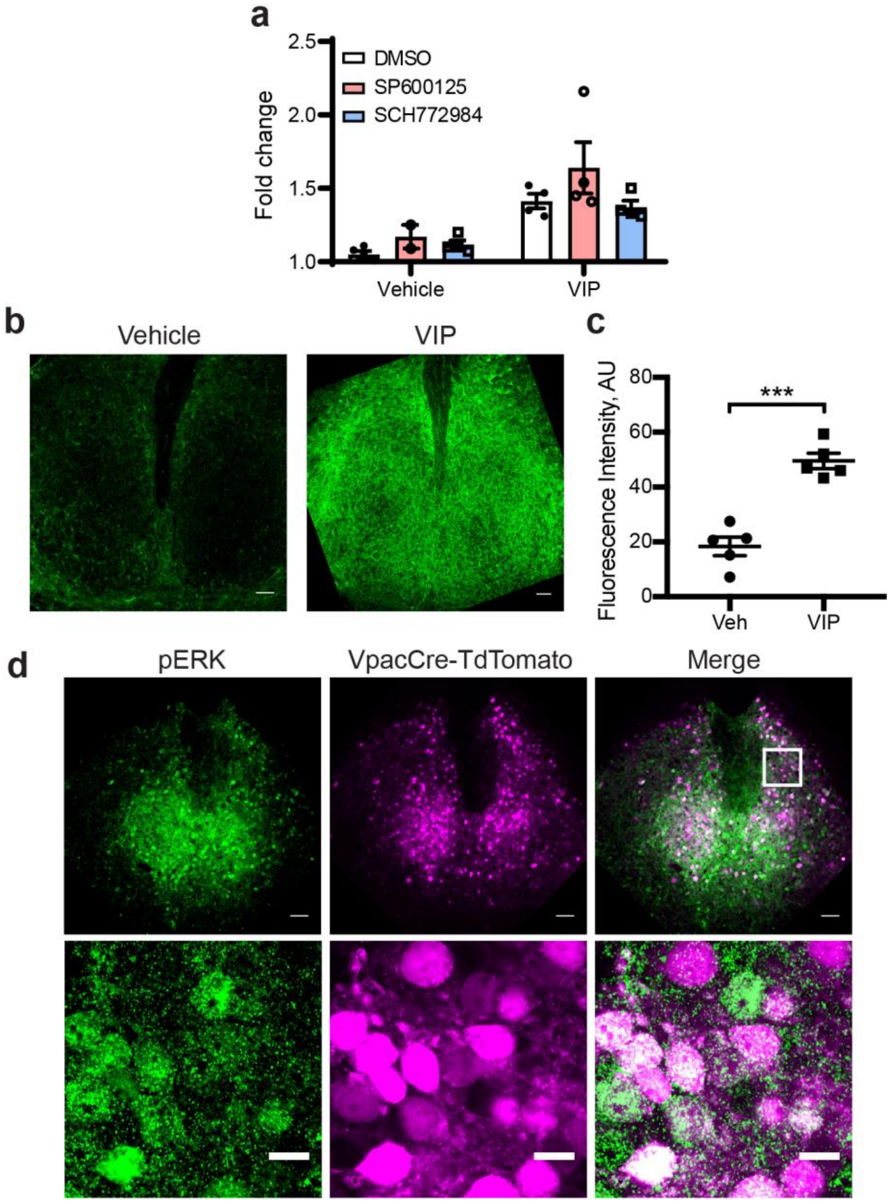

Supplementary Figure 6 | VIP acts through the phospho-ERK1/2 pathway. (**a**) Group data for acute fold induction of PER2::LUC after 3 µM SP600125 (JNK1/2/3 inhibitor), 100 nM SCH772984 (ERK1/2 inhibitor) or vehicle. (**b**) Representative confocal micrographs of SCN slices immunostained using an anti-phospho-ERK1/2 antibody following a 30-minute treatment with vehicle or 100 nM VIP at CT10. Scale bars represent 50 µm. (**c**) Group data for fluorescence intensity of SCN slices immunostained using an anti-phospho-ERK1/2 antibody following a 30 minute treatment with 100 nM VIP at CT10 as in (**b**) (mean ± SEM, veh, n = 5; VIP, n = 5; unpaired t-test, ***P < 0.001). (**d**) Confocal micrographs of a *VpacCre-TdTomato* SCN slice immunostained using an anti-phospho-ERK1/2 antibody following a 30-minute treatment with 100 nM VIP. Top: 20x. Bottom: 63x magnification of white boxed area. Scale bars represent 50 µm (top) or 10 µM (bottom).

Supplementary Figure 7

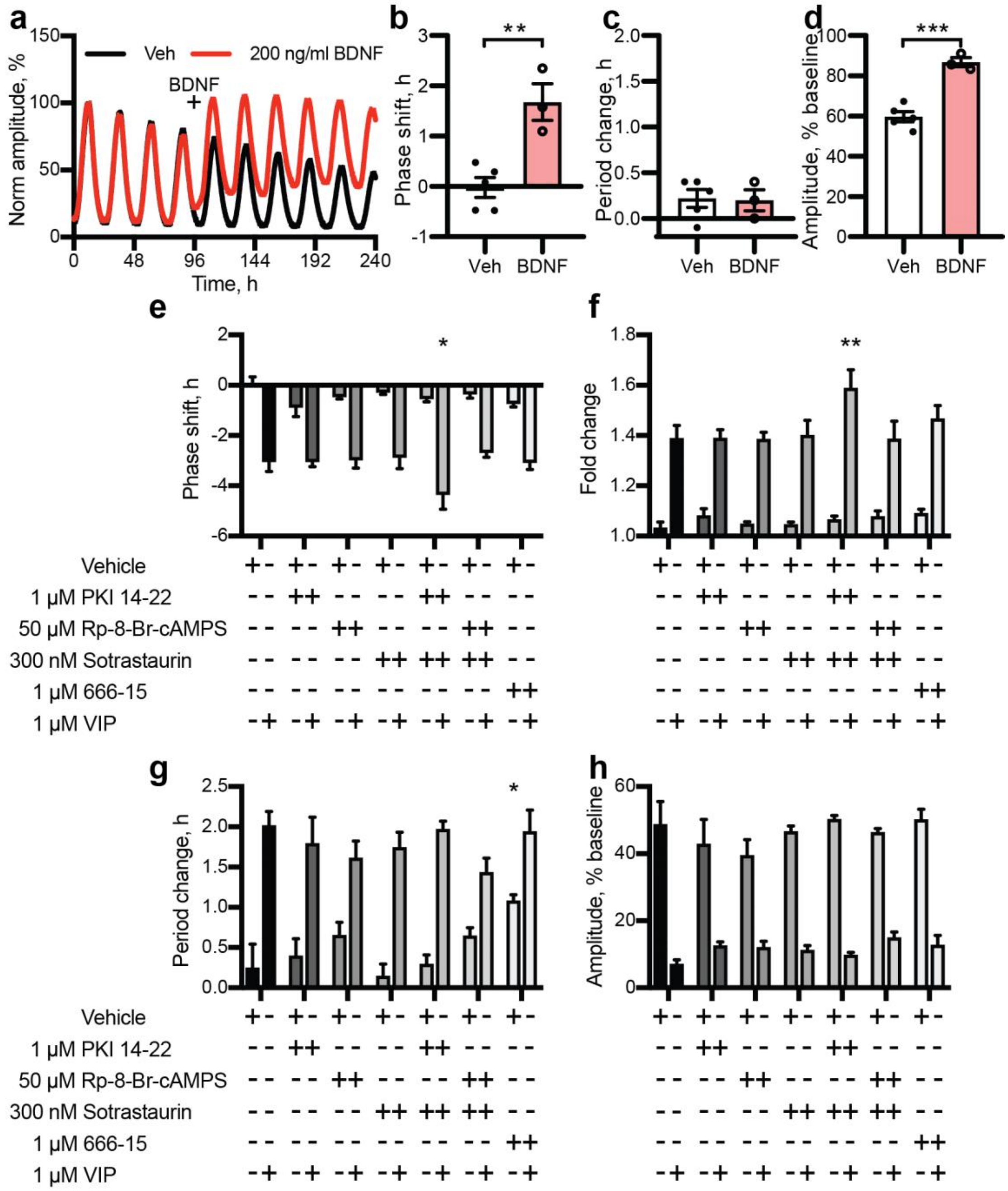

Supplementary Figure 7 | Pharmacological analysis of pathways necessary for transducing the effects of VIP on SCN circadian properties. (**a**) Representative PER2::LUC bioluminescence rhythms of SCN slices treated with 200 ng/ml BDNF or vehicle at CT16 (marked by +). Bioluminescence was normalised to the first peak. (**b-d**) Group data for phase shift (**b**), period change (**c**) and relative amplitude (**d**) responses (mean ± SEM, unpaired t-test, ***P* < 0.01, ****P* < 0.001) to BDNF (n = 3) or vehicle (n = 5). (**e-h**) Group data for phase shift (**e**), PER2::LUC fold change (**f**), period change (**g**) and relative amplitude (**h**) responses to VIP or vehicle in the presence of inhibitors of PKA (1 μM PKI 14-22 amide myristoylated; 50 μM Rp-8-Br-cAMPS), PKC

(300 nM sotrastaurin) or CREB (1 µM 666-15), alone or in combination as indicated. Two-way ANOVA with Dunnett's multiple comparisons test, n = 4-5 per group. * denotes significant difference to vehicle control. *$P < 0.05$, **$P < 0.01$, ***$P < 0.001$.

Supplementary Figure 8

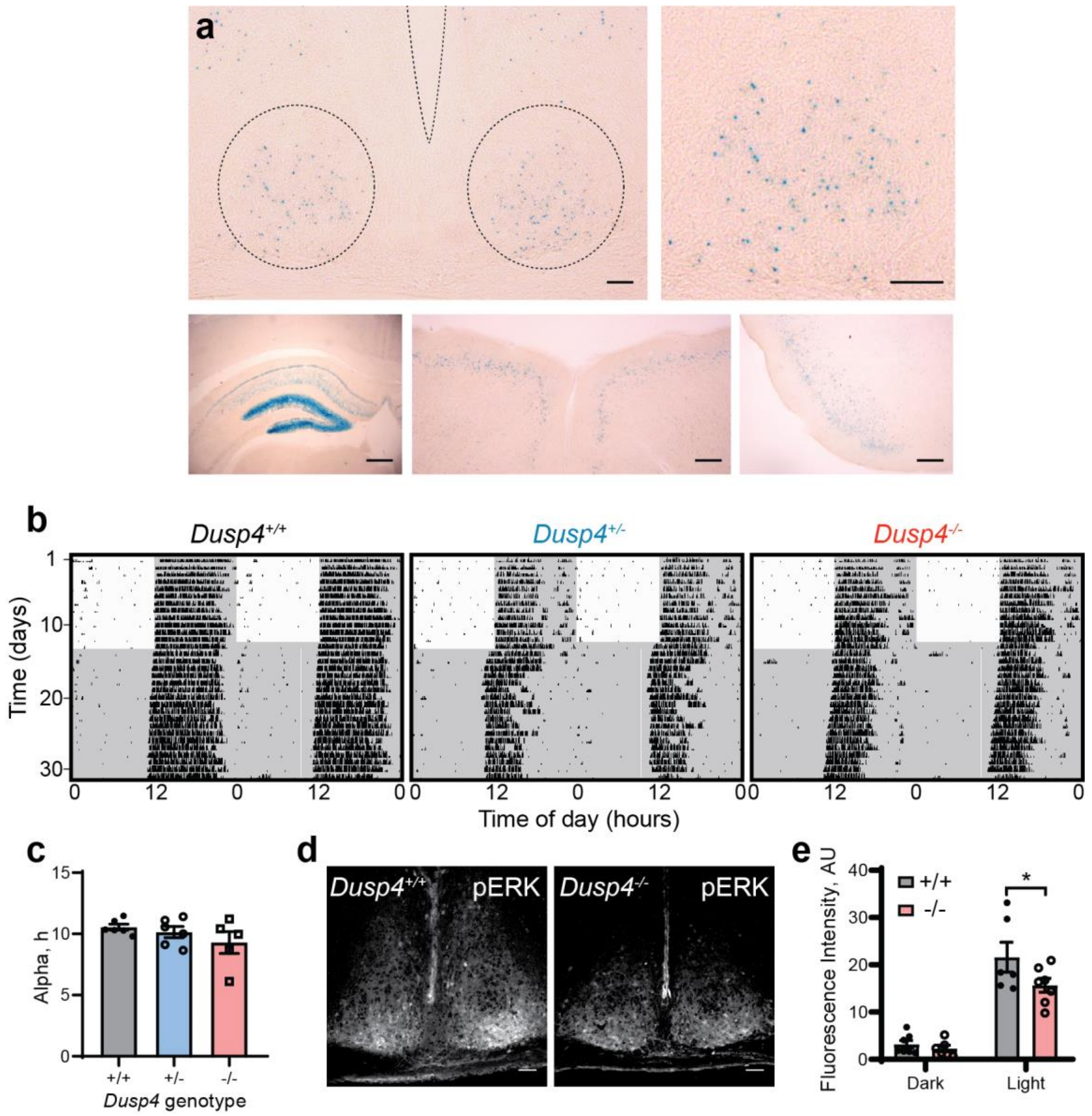

Supplementary Figure 8 | Characterisation of the global DUSP4$^{-/-}$ mouse. (**a**) Representative images of X-gal stained brain sections from mice homozygous for the DUSP4$^{-/-}$ allele, which contains a LacZ reporter gene inserted into the *Dusp4* locus. Staining was found in the SCN (top left, 10x, dotted lines delineate SCN and 3rd ventricle; close up top right, 20x), hippocampus (bottom left, 4x), cerebral cortex (bottom middle, 4x) and piriform cortex (bottom right, 4x). Scale bars represent 100 µm (top row) or 250 µm (bottom row). (**b**) Representative double-plotted actograms of wheel-running behaviour of *Dusp4*$^{+/+}$, *Dusp4*$^{+/-}$ and *Dusp4*$^{-/-}$ mice exposed to a 12:12 light:dark (LD) cycle followed by continuous dim red light (DD) conditions. Grey shading represents dim red light. (**c**) Alpha (length of time between activity onset and offset) of *Dusp4*$^{+/+}$ (n = 6), *Dusp4*$^{+/-}$ (n = 6) and *Dusp4*$^{-/-}$ (n = 5) mice (mean ± SEM) in DD. One-way ANOVA with Dunnett's multiple comparisons test. (**d**)

Representative confocal micrographs of SCN sections from *Dusp4*$^{+/+}$ and *Dusp4*$^{-/-}$ mice immunostained using an anti-phospho-ERK1/2 antibody following a 30-minute light pulse at ZT14. Scale bar represents 50 µm. (**e**) Group data for fluorescence intensity of SCN sections immunostained using an anti-phospho-ERK1/2 antibody following a 30 minute light pulse at ZT14 as in (**d**) (mean ± SEM, n = 6-7 mice per group). Two-way ANOVA with Sidak's multiple comparisons, $*P < 0.05$.

Supplementary Figure 9

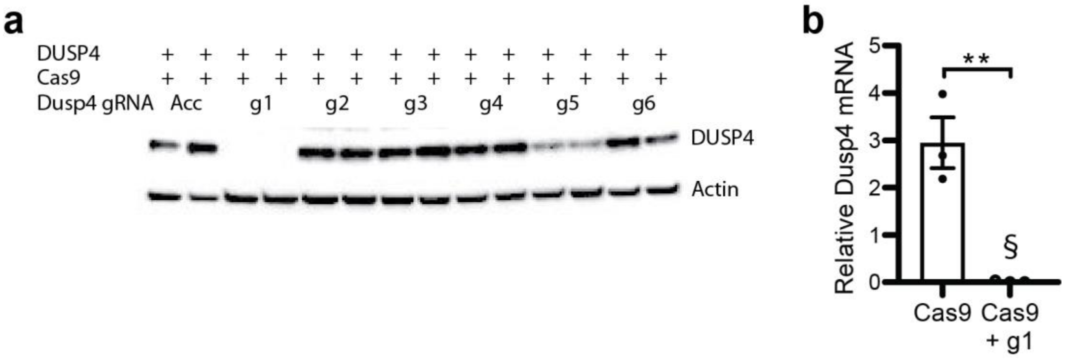

Supplementary Figure 9 | Validation of CRISPR-mediated knockout of Dusp4. (**a**) Representative Western blot using an anti-myc antibody for myc-tagged DUSP4 following transfection. N2A cells were transfected with *CMV.Dusp4-myc*, *Mecp2.SpCas9* and a gRNA construct designed against *Dusp4* (or the original gRNA 'acceptor' plasmid template, Acc), and harvested 72 h later. Actin serves as a loading control. n = 2 wells per condition. (**b**) Relative *Dusp4* mRNA expression (mean ± SEM, n = 3) assessed by qPCR in SCN slices following transduction with AAVs encoding *Mecp2.SpCas9* alone or with *U6.Dusp4g1.hSyn.GFP-KASH*. Expression levels were normalised to RNS18 levels to account for total RNA differences. §: When transduced with both Cas9 and g1, no *Dusp4* expression could be detected in 2/3 samples, while the 3rd had very low levels (relative expression: 0.1). Slices were harvested at CT6 (peak of *Dusp4* mRNA expression).

Supplementary Figure 10

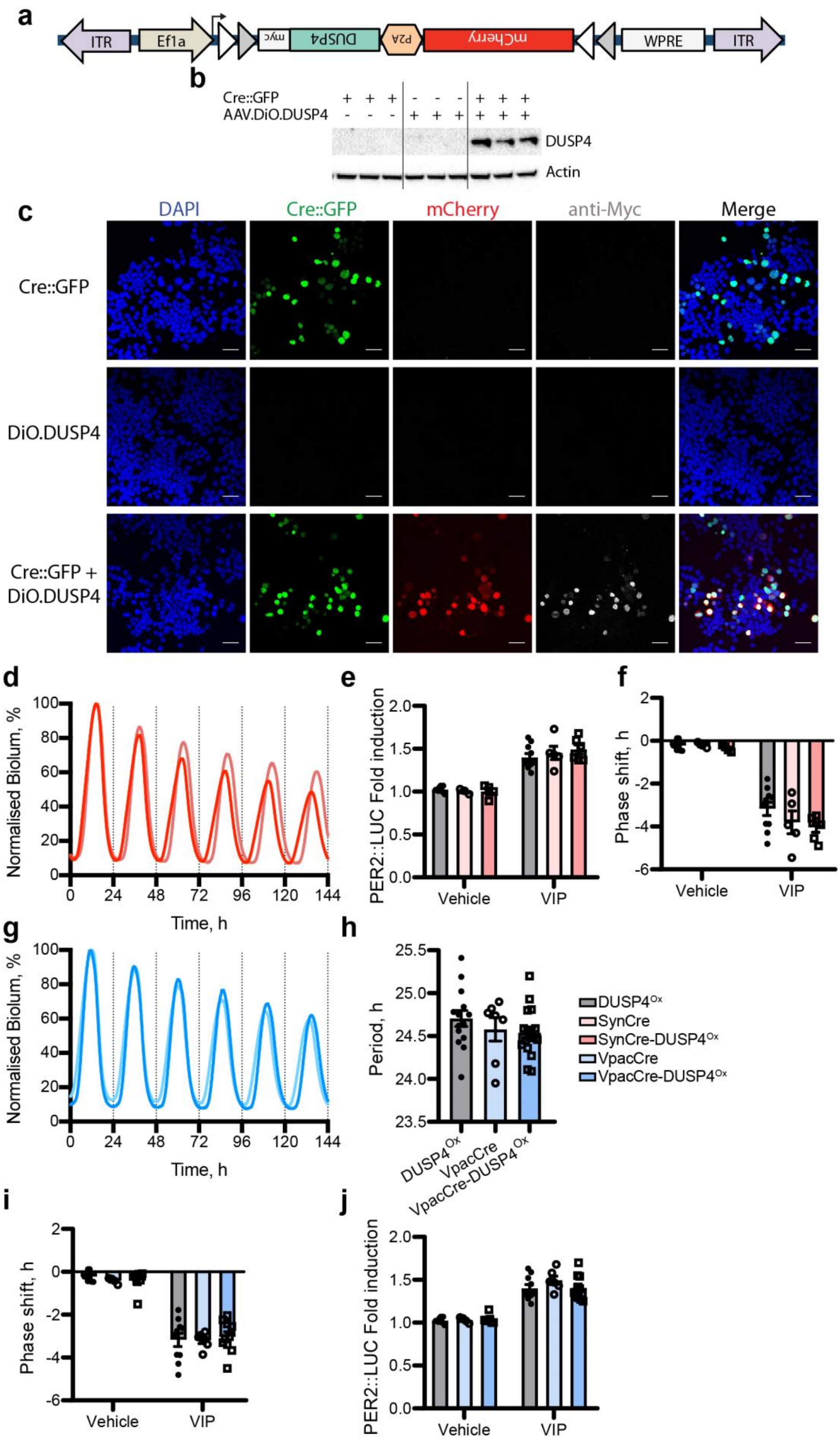

Supplementary Figure 10 | Validation of Cre-mediated overexpression of Dusp4. (**a**) Construct map of *Ef1a.DiO.mCherry-P2A-Dusp4.* DUSP4 and the mCherry are in a double-floxed inverse orientation (DiO). (**b**) Western blot using an anti-myc tag antiserum for myc-tagged DUSP4 following transfection. N2A cells were transfected with *Cre::GFP*, *Ef1a.DiO.mCherry-P2A-Dusp4,* or both plasmids. DUSP4 bands were only seen in the presence of Cre. n = 3 per group. (**c**) Representative images of N2A cells transfected as in (**b**) and immunostained using an anti-myc antibody. mCherry expression and myc-tagged DUSP4 can only be seen in the presence of Cre. Myc-tagged DUSP4 shows the expected nuclear localisation, compared to mCherry, which is throughout the cell. Scale bar represents 50 µm. (**d**) Representative PER2::LUC bioluminescence rhythms of *SynCre* and *SynCre-DUSP4*^{Ox} slices before treatment. Bioluminescence has been normalised to the first peak. (**e,f**) Group data for post-treatment phase shift (**e**), and PER2 induction (**f**) (mean ± SEM) of slices treated with vehicle or VIP at CT10. n as follows: *DUSP4*^{Ox}: veh, 8; VIP, 10; *SynCre*: veh, 3; VIP, 5; *SynCre-DUSP4*^{Ox}: veh, 4; VIP, 6. (**g**) Representative PER2::LUC bioluminescence rhythms of *VpacCre* and *VpacCre-DUSP4*^{Ox} slices before treatment. Bioluminescence has been normalised to the first peak. (**h**) Group data for steady state (pre-treatment) period of slices transduced as in (**g**), along with *DUSP4*^{Ox} controls (mean ± SEM, one-way ANOVA, n = 14 for *DUSP4*^{Ox} group, n = 7 for *VpacCre*, n = 16 for *VpacCre-Dusp4*^{Ox}). (**i,j**) Group data for post-treatment phase shift (**i**), and PER2 induction (**j**) (mean ± SEM) of slices treated with vehicle or VIP at CT10. *DUSP4*^{Ox} data replicated from (**e,f**). n as follows: *VpacCre*: veh, 4; VIP, 6; *VpacCre-DUSP4*^{Ox}: veh, 7; VIP, 10. All tests in (**e,f,i,j**) were two-way ANOVAs with Tukey's multiple comparisons. *$P < 0.05$, **$P < 0.01$, ***$P < 0.001$.
