# Supplementary material for: Vasoactive intestinal peptide controls the suprachiasmatic circadian clock network via ERK1/2 and DUSP4 signalling
Source: Nat Commun. 2019 Feb 1;10:542. doi: 10.1038/s41467-019-08427-3 (PMC6358603; doi:10.1038/s41467-019-08427-3)
Supplement: Supplementary file 3 — Description of Additional Supplementary Files [file 41467_2019_8427_MOESM3_ESM.pdf]

Description of Additional Supplementary Files

File Name: Supplementary Movie 1
Description: PER2::Luciferase bioluminescence recording from SCN slice before and after treatment with VIP.

File Name: Supplementary Movie 2
Description: Simultaneous recordings of CRE::Luciferase bioluminescence and RCaMPfluorescence reporting intra-cellular calcium from SCN slice before and after treatment with VIP.
